# Equine-assisted interventions for veterans with posttraumatic stress disorder: a systematic review

**DOI:** 10.3389/fpsyt.2023.1277338

**Published:** 2023-11-03

**Authors:** Jiaxin Li, Raúl Sánchez-García

**Affiliations:** Facultad de Ciencias de la Actividad Física y del Deporte (INEF), Universidad Politécnica de Madrid, Madrid, Spain

**Keywords:** equine-assisted interventions, post-traumatic stress disorder, veterans, systematic review, equestrian

## Abstract

Equine-assisted intervention therapy has a nearly 60-year history and has been shown to have a significant positive impact on various types of psychotherapy patients. Due to an increase in research on EAT, the number of existing methods of equine-assisted intervention therapy has gradually increased. Based on existing literature on the application of equine-assisted intervention therapy on veterans with post-traumatic stress disorder (PTSD), this study examines the characteristics of several types of equine interventions and includes a systematic review of peer-reviewed literature on equine-assisted interventions for veterans with PTSD published over the past 5 years, from 2018 to the present. Ten articles met the review criteria and served as the primary data for analysis. Several types of equine-assisted interventions were shown to have a beneficial psychological impact on veterans. However, some limitations were also found in the studies, such as that the majority of experiments were constrained by small sample sizes. Equine-assisted intervention therapy has been shown to be effective, but further research is merited, in order to focus on the specific details and theories involved in equine-assisted interventions, and on the welfare of the horses involved in the therapy.

## 1. Introduction

Danish dressage rider Lis Hartel is credited with being the inspiration behind the field of therapeutic horse riding. She won a silver medal in Grand Prix dressage at the 1952 Olympics in Helsinki, Finland, despite being severely disabled by polio. As a result of this amazing achievement, medical and equine specialists in Europe began offering therapeutic riding programs at their facilities (1). The research into equine-assisted healing began in 1960 and spans more than six decades. The use of horses in therapy is growing and is considered a viable option for treating a wide range of mental health disorders (2).

According to reports in the available literature, there are 600 equine-assisted intervention programs used by the Equine-Assisted Growth and Learning Association (EAGALA) (3). Equine interventions have grown in popularity over the years (4). Up until the present, equine-assisted intervention therapy research addressing a variety of mental health problems, including post-traumatic stress disorder (PTSD), has been limited and often poorly constructed, characterized by small sample sizes, unreliable assessments, unstandardized treatment techniques, and conflicts of interest among researchers.

The high risk of trauma that can result from combat, injury, captivity, and sexual assault faced by military personnel increases the prevalence of PTSD from 10% (civilian) to 30% (military personnel) (5–7). As research subjects, military personnel with PTSD play a critical role in the field of PTSD research. According to current research, the participation of horses in treatments has evolved into a novel auxiliary treatment method for a variety of diseases.

Therefore, in this study, we examine previous treatment cases with PTSD-afflicted veterans, in order to provide a theoretical and empirical foundation for the advancement of therapeutic horse engagement. More specifically, the main objectives of the paper are to describe the demographics of the veterans who participated in equine-assisted interventions and the screening processes that were used in their selection, as well as to describe the specific characteristics of equine-assisted interventions that have been applied to veterans, including intervention methods, study design, and results.

As previously mentioned, equine-assisted interventions have been an integral part of animal-human interaction therapy for 60 years. Despite the fact that recent systematic (8) and narrative review (9) have been conducted in this field, the present review introduces a more comprehensive analysis of the five distinct kinds of therapies. Also, this systematic review presents the treatment procedures for veterans in the form of a table, providing a more intuitive representation of the unique characteristics of each therapy.

The structure of the paper is the following: The first section presents an introduction to several different equine therapy methods. The second section gives the details of these methods and discusses the current state of equine therapy research for PTSD over a 5-year period.

### 1.1. Description of PTSD

According to the Diagnostic and Statistical Manual of Mental Disorders (DSM) (10), the diagnostic criteria for PTSD can include one or more of four domains: the re-experiencing (recurring thoughts or dreams) of traumatic events, avoidance (avoiding thoughts or feelings related to traumatic events), negative thoughts and moods (blaming oneself and/or others and having a pessimistic outlook), and anger arousal (outbursts of rage). In addition, significant functional impairment, varying psychiatric comorbidities, suicidal tendencies, substance abuse, chronic pain, poor physical health, and delayed seeking of treatment are all linked to the long-term effects of PTSD (11–13).

PTSD is a stress and trauma-related disorder, in which symptoms develop after exposure to one or more traumatic events (14). Reduced quality of life, substance abuse, suicide, risky and unhealthy behaviors, decreased productivity, domestic violence, and impaired relationships are all risks faced by people with PTSD (15). Traumatic neurosis (related to an individual’s vulnerability) is not the root cause of PTSD; rather, an external (traumatic) event triggers the disorder. The research of Gillies et al., Bradley et al., and Schneier et al. show that while some participants experienced an improvement of symptoms during treatment, others did not (16–18).

### 1.2. Animal assisted interventions

Animal-assisted therapy (AAT) is a therapeutic method that involves a patient, a therapist, and a trained animal, with the goal of achieving a predetermined therapeutic objective (19). Animal-assisted therapies (AATs) have proven effective in treating people of all ages, including those with both mental and physical impairments.

AAT has been utilized effectively as a treatment method for adults and children with psychological and physical disabilities. It is an adjunctive treatment designed to benefit the patient’s affective, cognitive, motor, and social functions (20). It has been demonstrated that AATs can improve communication, patient responsiveness, social interactive skills, socialization, activities of daily living, and general well-being (21).

Several different species of animals can be employed in AAT, with cats, dogs, and horses currently being the most common (22, 23). It is also important to note that AAT has been used successfully to help treat a variety of physical diseases. There is a significant amount of literature on the topic of using equine-based alternative activity interventions (AAIs) on people who have experienced trauma (24). Despite the widespread use of AATs, there have only been a small number of high-quality empirical studies that have examined their efficacy in the treatment of PTSD.

#### 1.2.1. Equine-assisted interventions

Proponents of equine-assisted intervention argue that the therapeutic interactions between horses and humans can help patients gain new perspectives and alter their behavior by serving as catalysts for the emergence of new ideas and emotions. While the field of equine-assisted psychotherapy (EAP) is still in its infancy, there is substantial evidence in the literature supporting the use of AAT for people with trauma-related disorders. Some possible benefits are the development of nonverbal communication abilities through interaction with horses and a reduction of blood pressure, heart rate, and anxiety levels, and AAT can also effectively treat depression, anxiety, attention deficit/hyperactivity disorder, conduct disorder, dissociative disorder, Alzheimer’s disease, dementia, autism, and various other chronic mental illnesses (23–31).

#### 1.2.2. Equine-assisted psychotherapy

EAP was established in 1990 and rapidly expanded in both Europe and the United States (32). EAP studies may always have limitations due to the variable nature of the intervention and the settings in which it is delivered, but rigorous randomized controlled trials are possible for evaluating EAP treatments (33).

EAP is similar to other AATs in that it is characterized by a lack of empirical evidence but includes many of the same components and benefits. EAP and AAT operate in different ways. First, dogs and cats are commonly employed for AAT because they are affectionate family pets. Horses are different from dogs and cats in that they are extremely sensitive to their environment and must be taught to trust humans. They are particularly sensitive to the moods and behaviors of the people around them (34). EAP has been shown to be a powerful and effective tool for participants who struggle with fear, depression, anger, anxiety, and other emotional disorders (35).

#### 1.2.3. Equine-assisted activities and therapies

Over the past decade, the use of horses in the context of equine assisted activities and therapies (EAAT) has exploded in popularity, with over 66,000 children and adults (including over 6,200 veterans and active-duty military personnel) receiving assistance from one of the 800 certified member centers and 4,800 certified instructors (36).

Previous research in the field of EAAT has primarily been focused on physical rehabilitation, and the majority of peer-reviewed publications have reported on the effects of human-horse interaction (29, 37–41). The treatment programs focus primarily on riding horses, and several related areas of research have led to significant advances in the treatment of physical mobility disorders (42).

#### 1.2.4. Equine-assisted therapy

EAT is gaining popularity as a complementary and alternative treatment for PTSD. EAT is also known as equine-facilitated cognitive behavioral therapy. There has not been sufficient research on the efficacy, feasibility, or safety of EAT in the treatment of PTSD, and there are no well-detailed treatment manuals for providing EAT. Therapeutic horseback riding (THR) and EAT-PTSD therapy, designed specifically for PTSD patients, are among the branches of EAT researched in the 10 articles used in this study. THR is a branch of EAT whose research focuses primarily on children with autism spectrum disorder (28). In addition, THR is an essential EAT technique.

However, THR should not be confused with hippotherapy, in which physical, occupational, and speech therapists specifically work with horses to improve the functional abilities of the patient (43). Through groundwork interaction and THR, bonding between the patient and the horse is facilitated (44).

#### 1.2.5. Equine-assisted services

The term “equine-assisted services” (EAS) is used to describe a wide variety of AAIs designed to benefit human users (45). Due to the fact that EAS interventions are in the initial phases of scientific development, thorough investigations are generally insufficient, and there is no standardized intervention strategy, which causes difficulty in making comparisons and replicating studies (33). One of the 10 articles used in this study employed a new model of EAS intervention called Whispers with Horses (46).

## 2. Methods

This study presents a systematic review. Comprehensive searches were conducted in the US National Library of Medicine (PubMed, Bethesda, MD 20894, USA), Medical Literature Analysis and Retrieval System Online (MEDLINE), and Web of Science databases. These databases are renowned for containing articles of exceptional quality and reliability, offering robust bibliographic support. We identified original articles focused on horse therapy from the past 5 years, detailed by the search strategy in Table 1.

**Table 1:** Searching strategy in databases.

Search strategy	Description
#1	(Horse PTSD) OR (therapeutic riding PTSD)
#2	Hippotherapy PTSD
#3	(Equine assisted PTSD) OR (horse treatment PTSD)
#4	# 1 AND # 2 AND # 3

**Figure 1:**
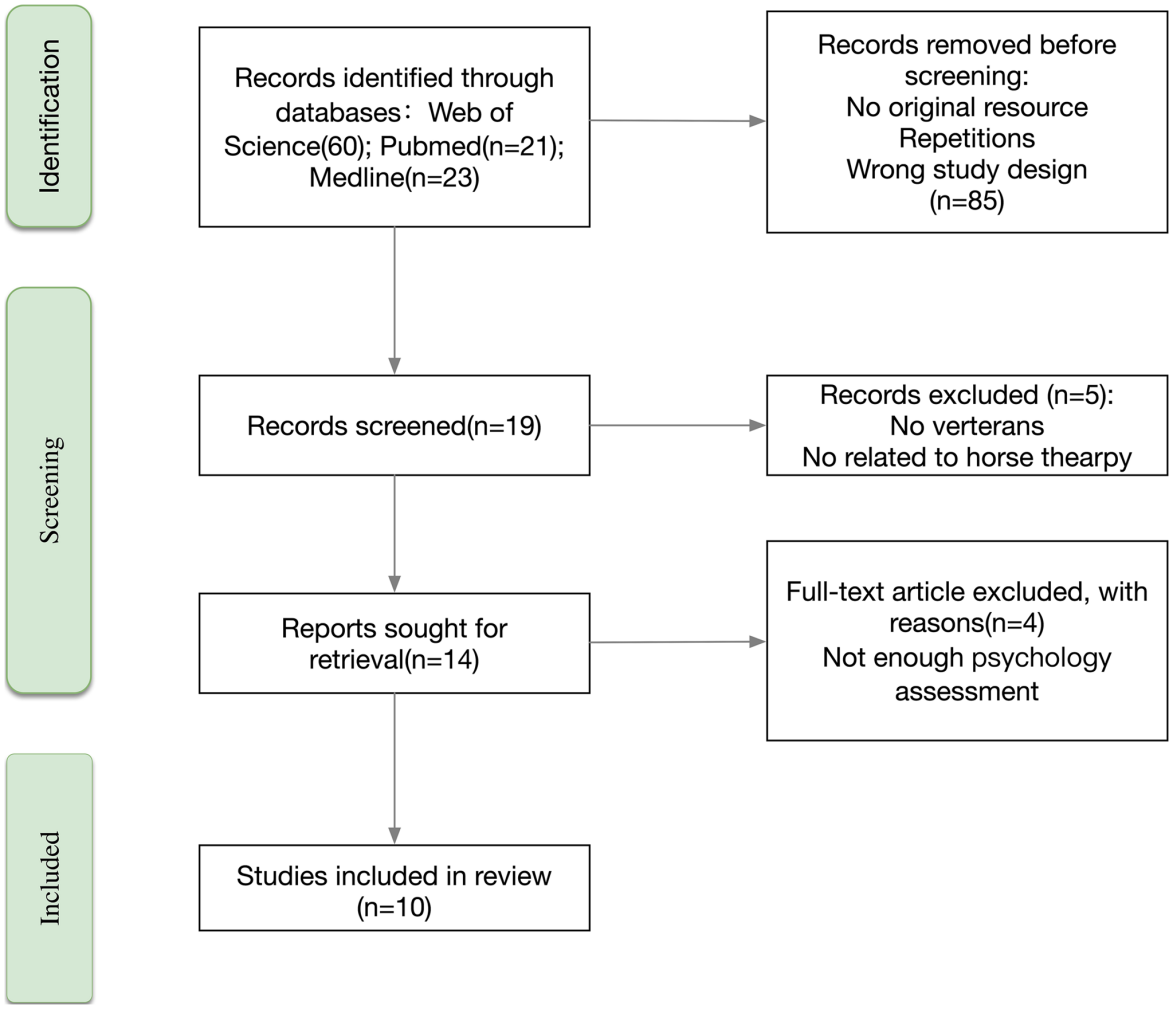
Methodology of selected articles.

A PRISMA flow diagram illustrating the search process is available (refer to Figure 1). The references from the selected studies were scrutinized to identify any additional pertinent articles. The most recent search for this systematic review was executed on 1 May 2023. There were no language restrictions.

Considering the emerging nature of the topic and the limited data available, no filters were applied concerning the study design; hence, both longitudinal and cross-sectional studies were considered. Similarly, there was no age limitation for the study populations. Two researchers (JL and RS) independently and concurrently performed the search, screened the titles and abstracts of 85 retrieved articles, assessed the full texts, and finalized the selection. From this process, 10 articles met the inclusion criteria.

Inclusion criteria specified original studies evaluating the role of live horses in Psychologically-Assisted Therapy for Humans, excluding interventions using horse simulators. Reports, letters to the editor, and other systematic and narrative review articles were not considered. Ultimately, 10 articles were included based on these criteria.

## 3. Results

In this descriptive review, we consolidated our primary findings into five tables. Table 2 details the 10 included studies, accompanied by information regarding their respective participants. Of these, eight were conducted in the United States, one in Israel, and another in Australia.

**Table 2:** Participates information.

Study	Country	Subjects	Sample size	Age
Shelef et al. (47)	Israel	Veterans with PTSD	23	28–48
Marchand et al. (33)	USA	Veterans with PTSD	18	28–69
Sylvia et al. (48)	USA	Veterans with PTSD and their family	106	25–57
Marchand et al. (49)	USA	Veterans with PTSD	33	46
Romaniuk et al. (50)	Australia	n = 25; Veterans only; Couples program (n = 22)	47	31–57
Arnon et al. (51)	USA	Veterans experiencing PTSD symptoms	8	30–61
Malinowsk et al. (36)	USA	Veterans with PTSD; Horses	7	31–68; horse age 10–23
Burton et al. (34)	USA	Veterans with PTSD	20	33–61
Fisher et al. (52)	USA	Veterans with PTSD	63	50
Johnson et al. (53)	USA	Veterans with posttraumatic brain injury, or both	20	29–68

Every veteran encompassed in the studies suffered from PTSD. A singular article delved into both PTSD and traumatic brain injury, whereas the remaining nine exclusively addressed PTSD. Each study specified age brackets, with participating veterans ranging from 25 to 69 years of age.

Table 3 shows the four equine intervention methodologies employed for the veterans. This table also elucidates the study design, assessment tools, veterans’ evaluation as research participants, post-intervention assessments, and the ensuing research outcomes.

**Table 3:** Investigation of equine-assisted interventions for veterans.

Study	Study design	Aim	Estimating Method	Subjects Assessment
Shelef et al. (47)	Open case study	To examine the impact of EAT on the symptons and functioning of individuals with PTSD.	Weekly frequency	A DSM-IV-TR diagnosis of PTSD for at least 1 year. Exclusion criteria included any orthopedic/neurological or other physical diseases that contraindicate equestrian riding, a concurrent psychosis diagnosis, and/or a reluctance to ride horses.
Marchand et al. (33)	Prospective cohort	To valuate a pilot program to treat PTSD using equine therapy in military personnel.	Weekly frequency	A comprehensive healthcare system administered by the Veterans Administration.
Sylvia et al. (48)	Thematic analysis	To research whether or not incorporating EAAT within a two-week intense therapeutic program for veterans with PTSD and/or TBI is feasible.	Weekly frequency	Satisfaction survey interview and questionnaire.
Marchand et al. (49)	Prospective open trial	To conduct the preliminary research necessary for future rigorous clinical trials of the Whispers with Horses intervention.	-	The subjects were recruited through referrals from the facility’s clinical staff. Participation in the study necessitated a history of military and/or civilian trauma. Active psychotic spectrum illness and/or cognitive impairment prohibiting meaningful participation were exclusionary criteria.
Romaniuk et al. (50)	A non-controlled, within-subjects longitudinal design	To examined the effects of an equine-assisted therapy program for Defence Force veterans and their partners on depression, anxiety, stress, posttraumatic stress, happiness, and quality of life. The difference between Individual and Couples program.	-	Included ex-serving Defence Force personnel or their partners, Mates4Mates members, and psychologist-approved program completers. Mates4Mates members must have served in the Australian Defence Force (ADF) and self-report a physical or psychological condition.
Arnon et al. (51)	Pilot open trial	To gauge the initial effects of EAT treatment protocol for PTSD (EAT-PTSD)	Weekly frequency	Telephone screening and questionnaire clinical referrals and print and online advertisements.
Malinowsk et al. (36)	A pilot field study	To measure plasma cortisol and oxytocin concentrations and HRV in horses participating in EAAT. To assess PTSD symptoms in previously diagnosed veterans before and after 5 days of EAAT, as well as heart rate and blood pressure readings during EAAT.	Daily frequency	All veterans with a history of Post-Traumatic Stress Disorder (PTSD) were recruited from Lakewood Veterans Affairs in New Jersey.
Burton et al. (34)	A two-arm, parallel group study design with delayed intervention in the control group.	To investigate how EAP affects PTSD symptoms.	Weekly frequency	Veterans with PTSD and a PCL-M score of at least 29 were eligible to participate. Adults who could not provide informed consent, children under 18, pregnant women, prisoners, antipsychotic patients, and glucocorticoid patients were excluded.
Fisher et al. (52)	An open trial of EAT-PTSD had 4 assessment points: pretreatment, midpoint, posttreatment, and 3-month follow-up.	To investigate if EAT-PTSD lead to potential therapeutic benefits for veterans.	Weekly frequency	Clinical referrals from Veterans Administration (VA) centers, other program affiliated with our center (the New York Presbyterian Military Family Wellness Centre at Columbia Veterans Research Centre), flyers, print and online advertisements, and word of mouth were utilized to close the gap in the table with the following study.
Johnson et al. (53)	A randomized clinical trial	To elucidate the experiences of veterans who participated in a 6-week THR program.	Weekly frequency	Electronic medical records; questionnaire

All 10 articles are underpinned by experimentally derived data. Broadly speaking, each article’s primary objective is to evaluate the efficacy of equine intervention as a therapeutic approach for veterans diagnosed with PTSD. Seven articles reported treatments administered weekly; one detailed interventions conducted on consecutive days; and two articles did not specify the duration of treatment but underscored its completion within the experimental timeframe. Out of the 10 articles, two explicitly indicated that PTSD diagnostic tests were conducted during participant recruitment, while the remaining articles relied on medical records for participant screening.

Table 4 delineates the specific interventions employed in each study, describes the data measurement methodologies, and summarizes both the findings and the identified limitations of these studies.

**Table 4:** Main findings.

Study	Intervention	Outcome measures	Overall findings	Limitation
Shelef et al. (47)	Group; EAT(equine-assisted therapy)	The Short Post Traumatic Stress Disorder Rating Interview (SPRINT)；The Sheehan Disability Scale (SDS) questionnaire	Improvements in daily functioning have been observed. This enhancement not only instills coping mechanisms but also fosters the development of a healthier, stronger self-image. Significant progress has been noted in functional domains, particularly regarding the ability to work and complete daily tasks as measured by the SPRINT scale. Additionally, there was a noticeable decrease in inefficiencies related to work, study, and household chores as per the SDS scale.	The sample size was small, resulting in limited statistically significant changes across all PTSD symptoms.
Marchand et al. (33)	Equine-assisted services (EAS)	Physical Activity Enjoyment Scale (PACES); Acceptance and Action Questionnaire II (AAQII); Positive and Negative Affect Scale (PANAS); Beck Depression Inventory (BDI-II); DSM 5 (PCL-V); Quality-of-Life Enjoyment and Satisfaction Questionnaire (QLES-Q-SF)	The AAQII may serve as a tool to investigate PF as a potential mechanism underlying the efficacy of this intervention. Despite not being a traditional mental health treatment, the intervention could offer psychological benefits to Veterans diagnosed with PTSD.	Initially, the study was uncontrolled, thus not delineating clear cause-and-effect relationships. The potential for selection bias was evident as randomization was not employed. The research faced challenges with subject attrition and a limited sample size, potentially limiting its generalizability to other Veterans. While PMM was utilized to impute missing data—a technique apt for nonparametric datasets—its application may not be suitable for studies with small sample sizes. Additionally, the fidelity of intervention delivery was not evaluated.
Sylvia et al. (48)	Equine-assisted activities and therapies (EAATs)	PCL-5: Posttraumatic Stress Disorder Check List for Diagnostic and Statistical Manual of Mental Disorders (Fifth Edition); PHQ-9: Patient Health Questionnaire; NSI: Neurobehavioral Symptom Inventory; AUDIT-C: Alcohol Use Disorders Identification Test.	Of the 62 seniors who participated in the program, 15 (24.2%) expressed interest in returning, while 13 (21.0%) opined that the weekend group size was optimal. Similarly, from the 44 families engaged in the program, 11 (22.4%) indicated their willingness to return, and 12 (24.5%) considered the weekend group size as ideal. Such findings provide initial evidence suggesting the acceptability of an adjunct EAAT program for veterans with PTSD and/or TBI participating in an IOP.	Rather than implementing a clinical trial, the study aimed to ascertain the feasibility of utilizing EAAT in veterans undergoing treatment for PTSD and/or TBI. The data were derived from anonymized satisfaction surveys.
Marchand et al. (49)	Group or individual; Whispers with horses – a model EAS intervention	PTSD Checklist for DSM 5 (PCL-V), the Patient Health Questionnaire 9 (PHQ-9), the Positive and Negative Affect Scale (PANAS), the Acceptance and Action Questionnaire II (AAQ-II), and the Physical Activity Enjoyment Scale (PACES).	Several sessions resulted in a significant enhancement in psychological flexibility, as evidenced by decreased AAQ-II scores, and demonstrated an improvement in affect, as reflected by PANAS scores. This trend might suggest that the efficacy of these sessions declines over time. Noteworthy reductions were observed in both PHQ and AAQ-II indices, indicating shifts in depression levels and psychological flexibility.	The study was limited by its small sample size. Furthermore, due to its uncontrolled design, causal relationships could not be definitively established. The lack of randomization also introduces potential selection bias concerns.
Romaniuk et al. (50)	Group; EAT	Depression Anxiety Stress Scale-21, Posttraumatic Stress Disorder Checklist for DSM-5, Oxford Happiness Questionnaire, and Quality-of-Life Enjoyment and Satisfaction Questionnaire-Short Form.	Throughout the duration of the program, participants indicated enhanced levels of happiness and life quality. However, these improvements were not sustained 3 months post-program. Notably, only participants in the Couples program sustained reductions in psychological symptoms at the three-month mark. This suggests that long-term psychological benefits may be more pronounced for couples compared to individuals.	The absence of a control group impedes definitive conclusions concerning the efficacy of the intervention. Outcomes might be influenced by uncontrolled variables, including involvement in other therapeutic interventions. Furthermore, a significant proportion of participants were not available for follow-up at the three-month interval.
Arnon et al. (51)	Group; Equine-assisted therapy (EAT)-PTSD	Diagnostic and Statistical Manual of Mental Disorders-5 (PCL-5 47), DSM-5; CAPS-IV; HAM-D; Beck Depression Inventory-II (BDI-II); Quality of Life Enjoyment and Satisfaction Questionnaire-Short Form (QLESQ-SF); Client Satisfaction Questionnaire (CSQ)	Post-treatment, five patients exhibited a response, with one achieving remission. At the three-month mark, three out of the initial five responders persisted in their response, whereas two regressed. Among the three individuals who did not respond post-treatment, one persisted in non-response, and two opted out of the follow-up assessment. A relapse was observed 3 months post-treatment. Of the seven individuals who did not achieve remission post-treatment, only one attained remission during the follow-up.	The sample size was limited and may not be representative. Patients concurrently undergoing psychotherapy or medication therapy were incorporated into the open trial, introducing potential confounding variables.
Malinowsk et al. (36)	Individual; equine-assisted activities and therapies (EAAT)	Brief Symptom Inventory and the PCL-5 (The PTSD Checklist for the DSM-5); Blood samples; Post-Traumatic Stress Disorder (PTSD);	An analysis of symptom clusters highlighted significant reductions in Cluster E Hyperarousal Symptoms. Concurrently, the PCL-5 Composite Score demonstrated marked reductions in PTSD symptoms, with heart rates showing a decline on day 2. Pertaining to horses: During EAAT, there was a noted decrease in the horses’ heart rates. No significant variation was observed in HRV variables such as SDNN and the LF/HF ratio. Additionally, plasma cortisol levels remained stable, suggesting the treatment regimen was not stressful for the horses. Furthermore, plasma oxytocin concentrations remained consistent in horses engaged in EAAT with veterans.	Measurement of the same hormones or physiological parameters for heart rate variability, as assessed in horses, was not feasible.
Burton et al. (34)	Group; Equine-assisted psychotherapy (EAP)	Salivary cortisol; PTSD Check List-Military Version (PCL-M); Connor-Davidson Resilience Scale (CD-RISC)	Participants reported enhanced levels of trust, relaxation, and patience. They also observed individual amelioration in symptoms and overall quality of life. EAP did not exhibit a significant alteration or increase in morning salivary cortisol concentrations.	Both the intervention and control groups suffered from a small sample size. Additionally, the study was limited by the lack of a randomization schedule. The research did not employ a dedicated instrument to gauge improvements in social or emotional functioning, or shifts in the overall quality of life. It is noteworthy that neither the intervention nor the control group excluded participants actively engaged in preexisting therapies. The study also did not consider the nature or quality of the participants’ previous professional therapies. Another limitation may stem from the short duration of the therapy sessions; EAP therapy was administered for a total of 6 h spread over 6 weeks, equating to 1 h per week.
Fisher et al. (52)	Group; Equine-Assisted Therapy for PTSD (EAT-PTSD)	PTSD Checklist for DSM-5 (PCL-5); (SCID-5-RV); Clinician-Administered PTSD Scale (CAPS-5); HDRS; self-report Beck Depression Inventory-II (BDI-II).	Reductions were observed in CAPS-5, PCL-5, HDRS, and BDI-II scores.	The absence of a control group without active therapy limits the study’s ability to evaluate the clinical efficacy of EAT, especially considering many participants were already on stable psychological and/or pharmacological treatments. Open trials may yield inflated results, and observed symptom changes could merely be attributed to the passage of time. Nevertheless, clinically significant symptom reductions were sustained at the 3-month follow-up. Furthermore, independent evaluators were not blinded to the open treatment, introducing potential bias. The study also did not account for any additional treatments received between the post-EAT assessment and the follow-up, as the intent was not to deter patients from seeking further treatment.
Johnson et al. (53)	Group; EAT-Therapeutic horseback riding (THR)	Coping Self Efficacy (CSES); traumatic brain injury (TBI); eight-item investigator-developed Riding Questionnaire.	Participants reported that THR fostered positive personal transformations, enhanced interpersonal connections among veterans, facilitated a bond between veterans and horses, and promoted constructive interactions between veterans and staff/volunteers. Additionally, the intervention was perceived to have minimal adverse effects.	The setting and timing of data collection possibly deterred veterans from giving comprehensive responses. The nature of survey environments can induce varied impacts. Although ensuring intervention fidelity complicated the study logistics, the co-authors achieved thematic consensus. However, this consensus does not guarantee participant concordance.

All 10 articles presented four unique equine interventions: five studies focused on EAT, two on EAS, two on EAAT, and one on EAP. Four of these studies employed questionnaires assessing quality of life, whereas seven used questionnaires specific to the diagnosis of mental disorders. Six articles conclusively established the efficacy of the equine intervention in symptom mitigation, but two did not yield definitive results. Within these inconclusive studies, two suggested potential treatment benefits, while the other two observed a resurgence of symptoms in the subjects 3 months post-intervention.

In terms of limitations, four studies highlighted their limited sample sizes. Sylvia et al. articulated that, rather than a clinical trial, their study sought to ascertain the feasibility of employing EAAT for veterans undergoing PTSD and/or TBI treatment (48). Marchand et al., across two distinct studies, emphasized that their research was not controlled and thus could not definitively establish causation (33, 46). Romaniuk et al. acknowledged the absence of a control group in their experiments (50). Fisher et al.’s study lacked an actively treated control arm (52), while Johnson et al. pointed out that environmental factors and data collection methods potentially influenced their experimental results (53).

Table 5 presents a descriptive overview of explanation and summary of how the equine-assisted interventions were executed across various studies.

**Table 5:** Explanation of horse intervention research.

Component	Description
Explanation of the planned activity	
Grooming	Grooming the horse.
Riding	Walking, trotting, cantering, steering, riding cross etc.
Break	During the session the horses have time to relax.
Groundwork	Groundwork includes actions like halting, turning, backing up, etc., as well as guiding the horse with or without a halter in a round enclosure or around an obstacle course.
Horse contact	Body language, facial expressions, and other forms of communication directed towards horses or people
Get to know the horse	Spending time with the horse, either in the pasture or the stable, observing it and getting to know it.
Matching horse and participant	In a deliberate pairing, either the student or the therapist/instructor choose the horse.
Horse care	Feeding, mucking stalls, and turning horses out and in from pasture.
Social activities	Increase activities that facilitate their social integration, such as group activities and life sharing.
Mounted exercises	Perform simple horseback exercises, such as stretching and balance training.
Prepare the horse	Prepare the horse for riding by installing the saddle, bridle, and girth, among other items.
Safety	During therapeutic rides, instructors adhere to these rules, including the use of protective gear. Additionally, safety is emphasized in the training.
Connections to daily life	Connecting ideas learnt through equine activities to the participant’s everyday life, maybe through dialogue or metaphor.
Family participation	Partners in the family or spouses took part in the session.
Integration of therapeutic practices	Equine intervention practice runs concurrently with other therapeutic modalities like cognitive behavioral therapy, mindfulness-based stress reduction, motivational interviewing, or reality testing.
Horse welfare	Mention about horse welfare.

Before initiating any experiment, participants were briefed about the treatment procedures and underwent preliminary exercises. Table 6 reveals that the average duration for the 10 EAT studies spanned 11 weeks, whereas EAAT treatments averaged a considerably shorter duration of 3.5 days. Every equine intervention approach necessitated more than 1 h per session.

**Table 6:** Equine-assisted interventions details.

Item	Shelef et al. (47)	Marchand et al. (46)	Sylvia et al. (48)	Marchand et al. (9)	Romaniuk et al. (50)	Arnon et al. (51)	Malinowski et al. (36)	Burton et al. (34)	Fisher et al. (52)	Johnson et al. (53)
Type	EAT	EAS	EAAT	EAS	EAT	EAT	EAAT	EAP	EAT	EAT
Session	24	4	3	6	-	8	5	6	8	6
Duration	6 months	4 weeks	Two-day, weekend	8 months	-	8 weeks	5 days	6 weeks	8 weeks	6 weeks
Time/session	3 h	1–2/4 h;3–4/2 h	2 h	90 min in group; individual 60 min.	-	1.5 h	1 h	1 h	1.5 h	1 h
Follow up	-	-	-	-	3 months	3 months	6 days	-	3 months	-
Explanation of the planned activity	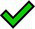 20 min	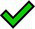	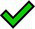	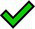	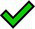	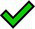	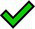	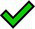	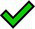	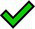
Grooming	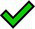	-	-	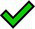	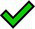	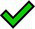	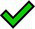	-	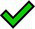	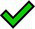
Riding	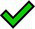 45 min	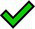 45 min cross	-	-	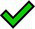	-	-	-	-	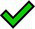
Break	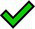 15 min	-	-	-	-	-	-	-	-	-
Groundwork	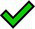 45 min	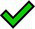	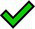	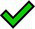	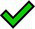	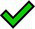	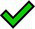	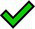	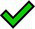	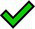
Horse contact	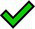	-	-	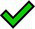	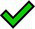	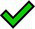	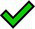	-	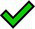	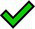
Get to know the horse	-	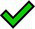	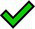	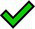	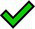	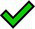	-	-	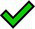	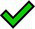
Matching horse and participant	-	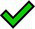	-	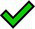	-	-	-	-	-	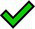
Horse care	-	-	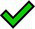	-	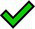	-	-	-	-	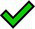
Social activities	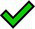 45 min	-	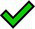 non-horse-based activities, such as quilting.	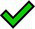	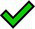	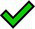	-	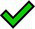	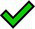	-
Mounted exercises	-	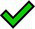	-	-	-	-	-	-	-	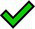
Prepare the horse	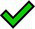 25 min	-	-	-	-	-	-	-	-	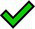
Safety	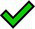	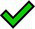	-	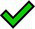	-	-	-	-	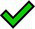	-
Connections to daily life	-	-	-	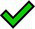	-	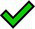	-	-	-	-
Family participation	-	-	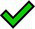	-	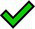	-	-	-	-	-
Integration of therapeutic practices	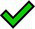	-	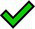	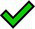	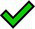	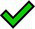	-	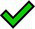	-	-
Horse welfare	-	-	-	-	-	-	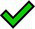	-	-	-

Three studies included a three-month follow-up evaluation, and another had a follow-up after just 6 days. As per Table 4, both Arnon et al. and Romaniuk et al. observed symptom recurrences and elevated test questionnaire scores after a three-month interval (23, 50, 52). Riding activities, with an emphasis on safety precautions, were featured in only four studies, three of which centered on EAT and one on EAS. Merely three out of the 10 studies ensured a compatibility match between the horses and the participants and also tended to the well-being of the involved horses. Two studies immersed participants in diverse activities, encompassing mounted exercises, horse tack routines, daily interactions, and family engagement. Six studies integrated specific therapeutic practices. Noteworthily, Malinowski et al. conducted simultaneous equine welfare assessments during their PTSD research, confirming that the treated horses remained unharmed (36).

## 4. Discussion

Preliminary data from the U.S. Army Medical Department suggest the potential benefits of animal-assisted therapy for wounded warriors participating in an occupational therapy life skills program. This underscores the significance of extended research in utilizing AAT as supplementary therapy for veterans diagnosed with PTSD and associated traumatic injuries (54).

The current review indicates that subjects showed discernible improvements in PTSD symptoms and a slight reduction in heart rate. Such findings align with established literature that details a multitude of potential physical and psychological advantages associated with animal-assisted interventions. In relation to EAAT studies that incorporated animals other than horses, the treatment duration was typically short-lived. A notable study elucidated that interacting or conversing with a dog led to reduced heart rates in participants. Within our reviewed research, the impact of EAAT involving horses on heart rate was especially prominent on days when veterans exhibited less activity and devoted more time to horse grooming and petting, as opposed to leading and roaming (55).

In studies utilizing EAAT with other animals apart from horses, treatment duration was typically brief. One study revealed that participants’ heart rates decreased when touching or conversing with a dog (56). The EAAT treatment with horses in the research we reviewed had an effect on heart rate, particularly on days when the veterans were more sedentary and spent more time grooming and petting the horses than leading and walking around (36).

Through a systems mapping analysis, we discerned that equine interventions could potentially benefit veterans with PTSD. A recurrent limitation in such studies, however, is the ambiguous details concerning the horses’ treatment within the research methodologies. While there are distinct breeds used for therapeutic purposes, the four horse-assisted therapies delineated in the summaries across these 10 articles exhibit no significant variation. Definitions across various categories also seem to converge.

Patients with PTSD face challenges in controlling emotions, maintaining reliance in relationships, and often harbor negative attitudes (57). Given these issues, the therapeutically value of equine-assisted interventions becomes evident, deriving from their attributes like fostering social relationships, sensory intentions, and their inherent inclusiveness and strength (58). Interacting with these huge animals provides patients both a sensory experience and a sense of control; this is further augmented by an experience-oriented approach that bolsters communication and mindful awareness during equine therapy (59). Through interaction with horses, patient benefit from emotional regulation and reflection (60). Apart from cognitive aspects, the experiential method of non-verbal embodiment combines physical and emotional dimensions, aiding emotional regulation, stress management, bolstering self-direction and resilience (61–63). Consequently, patients are better poised to re-establish trust. A review highlighted a statistically significant clinical improvement in symptoms after merely five 1-h sessions with horses (53). While traditional PTSD therapies can be prolonged, equine-assisted intervention therapy be a swift and effective method. Given that PTSD is a substantial predictor of suicidal ideation, the urgency for rapid-result treatments is essential.

Regarding equine welfare, horses engaged in therapeutic interventions encounter stressors, defined as environmental stimuli triggering homeostatic imbalances. Such stimuli elicit behavioral alterations, diminished immunity, and activations of the hypothalamic–pituitary–adrenal (HPA) axis alongside the autonomic nervous system (64). Chronic activation of any neuroendocrine axis can compromise equine welfare. The detrimental impact of a stressor hinges not on its intrinsic properties (intensity, duration, frequency) but on the predictability and controllability for the horse.

A specific horse study indicated that seasoned and rodeo-familiar horses manifest lower cortisol levels compared to their less experienced counterparts (65). The horses in our reviewed studies did not exhibit heightened fitness. Glucocorticoids have been prevalently employed as welfare indicators in animal welfare research (64). During EAAT sessions, horses exhibited reduced heart rates; metrics such as the standard deviation of normal-to-normal R-R intervals (SDNN) and the sympatho-vagal balance (LF/HF) ratio remained stable, implying a non-stressful session. Notably, in contrast to infrequent human-horse experimental setups, horses in Malinowski and colleagues’ EAAT investigations appeared unaltered (36).

EAT was adapted to address PTSD, with a variant being THR, categorized as an EAT sub-type. Fisher et al. posited that EAT-PTSD is safe and generally well-received (52). It could potentially engage avoidant patients averse to structured treatments, thereby fostering receptivity to further interventions. Johnson et al.’s findings highlight that the THR method involves direct riding, leveraging the necessity for riders to engage core muscles to remain upright on the horse (53). This enhances physical activity, diminishes stress, bolsters coping self-efficacy, and augments potential social support opportunities, potentially ameliorating PTSD symptoms and enriching veterans’ mental well-being.

Of the reviewed articles, only Burton et al. applied the EAP intervention approach. However, based on the intervention descriptions, participants were not involved in combined riding and psychotherapy sessions, rendering this EAP variant less impactful compared to others.

Extensive studies have underscored the efficacy of EAS in mitigating depression, anxiety, and PTSD symptoms among veterans (33, 66–69). Marchand et al., based in a prominent medical center, conceived the EAS-Whispers with Horses intervention in a bid for standardization. This method amalgamates mindfulness and self-compassion into a six-session psychotherapy and horse (PIH) program tailored for traumatized veterans. The integration with other psychotherapies, however, warrants further empirical validation.

## 5. Conclusion

In this systematic review, we critically evaluated publications from the last 5 years relevant to equine interventions for veterans with PTSD. Across the board, regardless of the specific equine intervention applied, all veterans with PTSD exhibited benefits.

While the majority of the examined studies utilized the EAT method, EAAT, although less frequently adopted, demonstrated pronounced effects even over short durations. Both EAS and EAP methods were comparatively rare, but their effectiveness was evident. A conspicuous gap in the literature is the detailed description of these interventions, underscoring the need for comprehensive investigation. Detailed delineation of intervention methodologies is imperative, as it can further optimize equine welfare within the interventions.

Of note, only a minority of the studies reviewed incorporated follow-up assessments, pivotal for discerning the longevity of the treatment effects on veterans with PTSD. Given that symptom recurrence is not uncommon post-treatment, there is a dearth of research on this front. Future inquiries should prioritize discerning whether the therapeutic effects persist over time.

Treating patients with PTSD often spans several years. However, all studies in the review indicate efficacy within a 6-month period. Equine-assisted intervention emphasize emotional regulation, mental control, and the mitigation of negative attitudes. Given these facets, other mental disorders share similar traits. Hence, there’s optimism that equine-assisted intervention therapy may benefit a wider range of patients in the future.

Furthermore, the methodologies lacked extensive descriptions regarding the horses’ roles in the interventions. This oversight is significant; a thorough understanding of the equine intervention methodology is essential to evaluate its impact on treatment outcomes more comprehensively.

Our review was confined to English-language studies, potentially omitting relevant research in other languages. Conventional systematic reviews typically do not include a rigorous assessment of the included studies, and in alignment, we did not undertake such an evaluation. Additionally, a granular evaluation of each individual equine intervention study was not conducted.

## Author contributions

JL: Methodology, Resources, Validation, Writing – original draft. RS-G: Supervision, Validation, Writing – review & editing.
